# Perception of a Perpetrator as a Successful Person Predicts Decreased Moral Judgment of a Rape Case and Labeling it as Rape

**DOI:** 10.3389/fpsyg.2018.02555

**Published:** 2018-12-11

**Authors:** Boglárka Nyúl, Anna Kende, Márton Engyel, Mónika Szabó

**Affiliations:** ^1^Doctoral School of Psychology, Eötvös Loránd University, Budapest, Hungary; ^2^Social Groups and Media Research Lab, Department of Social Psychology, Eötvös Loránd University, Budapest, Hungary; ^3^Department of Personality and Health Psychology, Eötvös Loránd University, Budapest, Hungary; ^4^Institute of Intercultural Psychology and Education, Eötvös Loránd University, Budapest, Hungary

**Keywords:** rape myth acceptance, rape, blame, perpetrator’s identity, perpetrator’s responsibility, perpetrator’s success

## Abstract

Rape cases of celebrities and other influential figures have caught the public eye in recent years. Following the media attention to these cases, people made strong judgments either believing or doubting the victims. Even though some of these men were convicted, they tended to receive little jail time and continued to enjoy people’s sympathy, as in the case of the Hungarian national swimming-coach. We examined whether opinions about the coach’s rape were affected by rape myth acceptance (RMA) and the perception of the perpetrator as a successful person. We conducted two online surveys to reveal this connection at two different points. The case was still somewhat ambiguous at the time of data collection for Study 1 (*N* = 870) because the perpetrator denied it. However, Study 2 (*N* = 105) took place after the perpetrator admitted his crime. In line with our predictions, we found that in the uncertain context of Study 1, RMA and the perception of the perpetrator as a successful person predicted whether respondents labeled the incident as rape, and how the perpetrator’s reactions were judged morally. In the certain condition of Study 2, RMA continued to predict moral judgments, but it no longer predicted whether the incident was labeled as rape. These findings showed that in the evaluation of a rape case of a popular and powerful person, perception of the perpetrator’s success can affect the overall evaluation of the case based on the level of RMA. However, such a connection is more pronounced when there are still ambiguities regarding the rape. We therefore suggest that both RMA and the effect of the overall perception of the perpetrator are considered in rape prevention programs, because rape cases rarely appear as certain and unambiguous in the media.

## Introduction

Following the revelations of the Harvey Weinstein case and the related public outrage, perpetrators of sexual abuse faced more serious consequences, such as termination of contracts and damage to their public image. However, this has not been and is not always the case when it comes to sexual offense committed by famous people. Bill Cosby, Roman Polanski, and László Kiss (head coach of the Hungarian swimming team) are successful and admired people, despite the fact that they all committed sexual assault or rape. This is not to say that they were not affected by the consequences of their offense, but against popular belief, rape accusations do not always mean the end of the perpetrator’s career and popularity. These people stayed popular and successful despite broad public awareness of their sexual misconduct. This is all the more surprising, as people consider rape an extremely serious crime, and have strong negative attitudes toward sex offenders (Ferguson and Ireland, 2006). In our study, we examined why a famous person can get away with rape (regardless of the fact that others may be judged extremely harshly for committing the same crime).

### The Concept of Rape Myth

Statistics on rape is unreliable because of the rate of unreported cases. They nevertheless suggest that it is a prevalent phenomenon affecting the lives of millions of people worldwide. One out of five women experienced rape in her lifetime in the U.S., and 1.3 million women reported some type of rape in the year of the survey in a study from 2010 (Black et al., 2011, NISVS). A much lower, but still high prevalence was found in Europe: one out of 20 women experienced rape. However, this number does not necessarily imply that there are indeed fewer cases in Europe, but it rather reflects a higher rate of unreported cases, as most analyses suggest. Specifically, according to the estimations of European Union Agency for Fundamental Rights (2014), only 11 out of 100,000 report rape to the police and this number greatly varies across countries.

Whether or not a victim of rape reports the case to the police is influenced by both personal and societal factors. The victim of rape may be reluctant to report it because of experiencing guilt, shame, embarrassment, fear of retaliation, and a lack of trust in the police (Sable et al., 2006). These emotions arise from the stereotypes about seductive and vindictive women that are also often used by defense attorneys to prejudice juries against the victim (Sable et al., 2006). Rape is often depicted as less violent or serious than it actually is (Yamawaki, 2007; Newcombe et al., 2008), testimonials of rape victims are often doubted and the psychological harm is underrated (e.g., Yamawaki, 2007).

These personal feelings are strongly connected to socially shared beliefs about rape: the so-called rape myths. Rape myths are “descriptive or prescriptive beliefs about rape (i.e., about its causes, context, consequences, perpetrators, victims, and their interaction) that serve to deny, downplay or justify sexual violence that men commit against women (Bohner, 1998, p. 14).” Rape myths either blame the victim (e.g., if a girl acts like a slut, eventually she is going to get into trouble) or excuse the perpetrator (e.g., rape happens when a guy’s sex drive goes out of control), rationalizing the otherwise highly uncomfortable thought that innocent people can be raped (Burt, 1980; Payne et al., 1999).

The importance of rape myth acceptance (RMA) is that it correlates with committing rape (Lonsway and Fitzgerald, 1994; Murnen et al., 2002). Relatedly, men are more likely to accept rape myths (Payne et al., 1999; Aosved and Long, 2006). Furthermore, those who accept rape myths are also more likely to identify women’s friendly behavior as sexually teasing (Haworth-Hoeppner, 1998; Willan and Pollard, 2003), and less likely to help rape victims (Foster and Kidd, 2014), suggest rape victims to report the rape (Frese et al., 2004), and label forced sex as rape (Burt and Albin, 1981; Norris and Cubbins, 1992; Peterson and Muehlenhard, 2004). Women’s RMA level determines whether they see rape as something that could happen to them, and whether exposure to rape cases lowers their self-esteem (Bohner et al., 2009). RMA can therefore function as a form self-protection which reduces anxiety about becoming a victim (Bohner and Lampridis, 2004). It can therefore comfort both women and men because it helps maintain the belief that they will neither become victims, nor perpetrators of rape.

Rape myths and RMA function as social norms as well. In previous research men’s rape proclivity was affected by perceived RMA of others, and the effect was moderated by the participant’s own RMA (Eyssel et al., 2006; Siebler et al., 2006; Bohner et al., 2010). Similarly, participants who read an article about rape with information based on rape myths were less likely to believe that the perpetrator was guilty than those who read an article with rape myth challenging information (Franiuk et al., 2008a). Rape myths presented in the media can increase their acceptance, especially among those who already endorse them. Media reporting that relies on rape myths also communicate their acceptability toward people who are otherwise not aware of them (Franiuk et al., 2008a). Although the general acceptance of overt rape myths has diminished over the years because of higher awareness and changing social norms, they continue to exist in more subtle forms (McMahon, 2007). In sum, RMA should be considered both in terms of individual differences and as the normative context of rape, as they both influence the threshold of labeling a case as rape, blaming a victim for the act, and considering the perpetrator guilty.

The concept of rape myths was first used in the 1970s (Schwendinger and Schwendinger, 1974; Brownmiller, 1975), and defined as cultural beliefs about sexual assault that support and trivialize male sexual aggression against women. By looking at commonly held responses to sexual assaults, Burt (1980) emphasized that the cultural function of rape myth is to normalize sexual violence and victim blaming, and relied in these responses in developing a measure of RMA (Rape Myth Acceptance Scale, RMAS). Lonsway and Fitzgerald (1994) pointed out the limitations of Burt’s (1980) scale, and developed a newer scale (Illinois Rape Myth Acceptance Scale IRMAS, Payne et al., 1999) that could explain the psychological mechanisms of victim blaming and its social consequences at the same time (Lonsway and Fitzgerald, 1994). As rape myths and its public expression became more subtle, researchers had to use more subtle scale items to measure its acceptance. Following these societal changes in the acceptance of blatant rape myths, McMahon and Farmer (2011) eliminated three subscales of IRMAS, updated its language, and reworded the items to capture the currently more prevalent subtle rape myths. In the current paper we examine these subtle rape myths, therefore we rely on the validated Hungarian translation of the McMahon and Farmer (2011) RMA scale (Nyúl et al., 2018).

### The Social Cognitive Effects of Rape Myth Acceptance

Rape myth acceptance creates bias in information processing similarly to other cognitive schemas. Eyssel and Bohner (2011) found that irrespective of whether the information was about the victim or the perpetrator, the more information participants received, the stronger the effect of RMA was on blaming judgments. In the same study, participants with high RMA who were made to believe that they received additional subliminal information about a rape case, although they did not, felt more entitled to judge. Using eye-tracking technique, Süssenbach and colleagues found that RMA affected information processing and visual attention in rape related pictures: participants with higher RMA were more sensitive to rape myth consistent cues and processed them easier, they preferred information about the victim instead of the perpetrator, and shifted their visual interest form the potential perpetrator to the victim (Süssenbach et al., 2012, 2017).

Group membership of the perpetrator and the victim can also produce bias in how a rape case is perceived and evaluated (George and Martínez, 2002; Harrison et al., 2008; Bal and van den Bos, 2010; Masser et al., 2010; McKimmie et al., 2014). This can be explained by social identity theory suggesting that people are motivated to see members of their ingroup more positively than members of the out-group (Tajfel and Turner, 1979). Previous research has shown that people blame an out-group perpetrator more than an ingroup perpetrator (George and Martínez, 2002; Harrison et al., 2008; Bal and van den Bos, 2010), and blame an ingroup victim less than a victim belonging to an out-group (Harrison et al., 2008).

Perception of a rape case is also affected by how the actual case fits with stereotypes about rape. Evaluations depend on whether the case is consistent with the so-called “real rape” scenario in terms of the perpetrator’s and victim’s gender, the relationship between them (Bell et al., 1994), and the victim’s behavior (L’Armand and Pepitone, 1982; Wyer et al., 1985). A rape case is perceived as stereotypical (i.e., real rape) if the perpetrator is a stranger and a deviant person (Greenberg and Ruback, 1992), he uses a weapon or physical force during the rape (McGregor et al., 2000), the victim reports the rape and cooperates with the police without hesitation (Bongiorno et al., 2016). Participants were more likely to blame the victim and believe that it was not rape when the case was perceived counter-stereotypical, that is, when the victim did not fight against the perpetrator physically and did not cooperate with the police (Sheldon and Parent, 2002). Putting together the effects of stereotypicality and group belonging, Bongiorno et al. (2016) found that the perpetrator’s out-group membership did not affect the evaluation of a stereotypical rape case, however, the ingroup perpetrator was more likely to be excused and the victim blamed for the rape when the rape was counter-stereotypic.

Knight et al. (2001) found that perception of rape is influenced by the perpetrator’s celebrity status as well: famous perpetrators were evaluated more positively than non-famous ones. Furthermore, participants recommended shorter sentences, considered the perpetrators more reliable and thought that victims enjoyed the rape more if the perpetrators were celebrities. Success can be a direct outcome of social status but success can be gained by competence. These two sources of success may have different implications for lenience. In the former case, it has to do with social power, and in the letter case, it is connected to positive personal qualities and deservingness (for a similar distinction see Pica et al., 2017).

The perpetrator’s social status also affects jurors’ judgment according to a meta-analysis (Devine and Caughlin, 2014). Perpetrators with lower SES are more likely to be convicted than perpetrators with high SES. Perpetrators with higher SES are seen less blameworthy and they are assigned shorter sentences (Gleason and Harris, 1976; Osborne and Rappaport, 1985). However, occupational social status does not affect jurors’ verdict directly, but high SES perpetrators are perceived as having better potentials in the future (Loeffler and Lawson, 2002). A more recent study has found that a perpetrator’s low social status affected whether he was seen guilty in a rape case after alcohol consumption, but not after taking cold medicine (i.e., whether they were considered responsible for their state). However, this difference was not found when the perpetrator was a star athlete (Pica et al., 2017).

Labeling a case as rape by the victim is also subject to similar biases. Peterson and Muehlenhard (2004) found that rape victims with higher RMA were less likely to label their own experience as rape. Participants did not consider the behavior as sex (e.g., there was no penile penetration, penile penetration was short) in order to avoid thinking that they had been raped. Another study suggests that some victims do not label their experience as rape because they did not experience a strong negative emotional reaction after the event (Kahn and Mathie, 2000). Victims are also more likely to label a situation as sexual assault when it fits with the prototypical rape script (Turchik et al., 2009).

In sum, previous research suggest that people’s strongly negative opinion of rape is mostly valid for rape cases that are perceived as stereotypical, or in situations in which a biased positive perception of the perpetrator (e.g., being a celebrity or a member of the ingroup) or a biased negative perception of the victim (e.g., alcohol consumption) does not influence their judgment. In line with this, if the rape is inconsistent with the stereotypical rape scenario, victims are less likely to report (Campbell et al., 2001) and people are more likely to blame the victim. This is all the more serious as most rape cases are not stereotypical (e.g., Ministry of Justice Home Office and Office for National Statistics, 2013; European Union Agency for Fundamental Rights, 2014).

### The Context of the Research

In our study, we were interested in understanding the mechanisms of bias using the example of a rape case of a famous and popular swimming coach in Hungary. Swimming is an important and highly successful national sport, and therefore, it receives a lot of public attention. Swimmers and coaches are generally well-known and highly popular people, and often considered as national heroes. Although the scandal was recent, the rape was committed 55 years earlier. The perpetrator was a successful young swimmer at the time, and he did not know his victim who was an 18-year-old woman visiting the swimming-pool that day for recreation. The case was known to some people in his immediate surroundings – he was even shortly imprisoned for committing this crime at the time – but it only became known to the wider public in 2016 (for a description of the scandal see Anderson, 2016). László Kiss was already a talented swimmer at the time of the rape, and later he became not just a successful swimmer, but also one of the most successful swimming coaches. His pupils became Olympic and world champions. In sum, he was a widely respected person who received his reputation through hard work and competence. The fact that he earned his success and deserved people’s admiration was also often mentioned in the media in connection to his rape case. At the time of the rediscovery of his rape, the swimming coach denied it. This evoked mixed reactions among Hungarians. Both media and social media reactions used rape myths to defend him and make his story credible (e.g., news reports suggested that the victim liked sex or she should not have had gone with the perpetrator to his apartment). Others expressed their disappointment in a popular and well-known person.

This case seemed suitable for examining the effects of a biased perception of the perpetrator on a rape case because the event happened a long time ago. As the victim was believed to be dead at the time the scandal broke out, many aspects of the rape were unclear. We found this case especially interesting because it had the potential to reveal whether a rape case was evaluated through the distorting lens that focuses on the perpetrator’s success even though the rape happened before he became a well-known and popular person. Thus, we could examine the effect of RMA and the related biased perception of the perpetrator more clearly than studies that focused on the evaluation of rape cases committed by people who were celebrities at the time of the rape. These studies have found that not only famous perpetrators were found less guilty, rape itself was seen differently (e.g., Knight et al., 2001).

Looking at a real-life event, we had the opportunity to identify the mechanisms of bias in the actual social context in which rape cases are evaluated and interpreted. This is especially relevant in the normative context of Hungary where reported rape cases are very low (European Union Agency for Fundamental Rights, 2014). Studies suggest that unreported cases are 415 times the reported cases (Wirth and Winkler, 2015). Hungary holds the 101st position in the global gender gap index regarding the equality of the positions of men and women in society (World Economic Forum, 2016). Although, we have no direct empirical evidence that this creates an environment in which victim blaming is more acceptable than in other social contexts, but the high rate of unreported rape suggests that victims do not feel a general support and that their stories would accepted as real (Sable et al., 2006; Wirth and Winkler, 2015) Therefore, the social costs of committing rape is not necessarily high.

The importance of understanding the connection between RMA and biased perception in the case of a real-life case is twofold. Firstly, it can provide information about the evaluation of cases in the complex social reality where judgments are affected by multiple factors in contrast to the controlled setting of the lab. Secondly, scandals that people talk about for weeks can strongly influence the normative context in which all other rape cases are evaluated. Journalists are not immune to the cultural context and victim blaming either. Researchers found that journalists were more likely to question the victim’s story than the perpetrator’s in their reports on rape cases, and 65% of the newspaper articles referred to a rape myth (Franiuk et al., 2008a). Victim blaming is also commonly found in newspapers (Los and Chamard, 1997; Korn and Efrat, 2004). Understanding these biases in reporting about rape is underlined by a study showing that people who read an article that endorsed rape myths were less likely to believe that Kobe Bryant (a basketball player who was accused of rape) was guilty than those who read an article with rape myths challenging thoughts (Franiuk et al., 2008a). Our research can potentially explain why social reactions to this particular highly publicized rape scandal were mixed, and it can also provide guidelines for the media on how to communicate rape cases.

### Hypotheses

Related to the case of the swimming coach, we examined whether the evaluation of the rape was affected by the perception of the perpetrator along traits that were irrelevant from the perspective of the case, such as being a successful swimmer or swimming coach (i.e., considering success an important factor in making judgments about the rape), as well as by individual differences in RMA. Specifically, we hypothesized that RMA would predict a higher importance of the perpetrator’s success in labeling the case as rape (in line with Eyssel and Bohner, 2011; Süssenbach et al., 2012) and in the moral judgment of the reactions to the rape case, such as its denial by the perpetrator. We also hypothesized that RMA would directly predict labeling the case as rape (Eyssel and Bohner, 2011). Rape is considered morally wrong and as a serious crime. Consequently, people have negative attitudes toward sex offenders (Ferguson and Ireland, 2006). Therefore, we hypothesized that rape labeling and moral judgment would be positively associated, that is, those who label the case more as a rape would have stronger moral judgments about the perpetrator. Finally, because RMA creates bias in information processing that affects the importance of different cues to participants regarding a rape case, we hypothesized that the importance of the perpetrator’s success – as an opportunity to excuse the perpetrator – would not be an independent variable from RMA, but mediate the connection between RMA and labeling the case as rape (see e.g., Knight et al., 2001; Loeffler and Lawson, 2002; Eyssel and Bohner, 2011; Süssenbach et al., 2012; Devine and Caughlin, 2014). Although there is evidence that the level of reported RMA depends on perceived social norms (Bohner et al., 2006), therefore, it could theoretically treated as an outcome variable in the context of perceived success of the perpetrator, previous research predominantly treated RMA as a stable construct. For this reason, we examined how RMA predicted irrelevant but absolving information about the perpetrator rather than how perceiving the perpetrator’s success in the context of the rape predicted the RMA level.

## Materials and Methods

### Study 1

#### Participants

Participants were recruited in two different ways. First, we collected data from a self-selected community sample (*n* = 504; 19.6% male, *M*_age_ = 37.52, *SD*_age_ = 12.34) using convenience sampling by posting the link of the questionnaire on Facebook in various women’s groups including groups with a clear focus on violence against women. This method of sampling reached respondents who were likely to be motivated to express their opinion about the case. Their opinion is not representative to the public opinion, nevertheless relevant to consider as they are motivated to influence the public perception and public debates about a topic. However, in order to create a more balanced sample, we also collected data amongst university students, to include people with less established attitudes about rape and rape myth to the sample. Students received credit points for participation (*n* = 366; 25.1% male, *M*_age_ = 21.33, *SD*_age_ = 1.84). The final sample size was *N* = 870.

#### Procedure

We used an online questionnaire and conducted the study following the IRB approval of Eötvös Loránd University. We report all data exclusions and measures that are relevant to the research question in this study.

Participants were presented with a summary of the scandal without any evaluations. The description contained only the dates of the event and its recent discovery, as well as the reactions of the National Swimming Association (which was defensive of the coach). As the case was recently discovered, and few people knew about it from the time that it happened, we did not ask people to recall the events or whether they knew about it before the current scandal broke out, but only asked them to offer their opinion about the case assuming that they learned about it in the present and were reminded of its details in the short description that we provided. Nevertheless, respondents may have been influenced by different interpretations of the events by their exposure to different media sources. It was for this reason that we launched our questionnaire shortly after the scandal broke out, and before opinions could have been crystallized pro or contra the case. We started collecting data 9 days after the scandal broke out when it was still a widely discussed topic in mainstream and in social media. Based on Google search more than 160 online newspaper articles included the key words “László Kiss” and “rape” between 5/4/2016 and 13/4/2016, that is, between the first article discussing the case and the start of our data collection.

#### Measures

Participants indicated the importance of the perception of the perpetrator’s success in their evaluation of the rape case by two items (How important is it that he was a successful swimmer? and How important is it that he was the swimming coach of the Hungarian national swimming team? *r* = 0.76, *p* < 0.001) on a 7-point scale (1 = completely unimportant, 7 = very important). As we have mentioned in the introduction, success can be achieved through a person’s social status or it can be the result of hard work and discipline. In this case both interpretations could be applicable, because he did not only hold a high social status by being the national swimming coach (i.e., being in a position of power), but also earned his position through a lifetime of hard work. While both of these perceptions can have the same distorting influence on the evaluation of the rape case through perceptions of deservingness, we were not particularly interested in measuring the actual perception of the perpatrator’s success, but only its importance to the participants in the context of the rape. To put it differently, we measured the extent to which participants evaluated the rape case in light of the perpetrator’s perceived success.

We measured the moral judgment of the perpetrator’s response by four items that we created for the purpose of this questionnaire. We asked respondents to evaluate whether the following responses were morally right: [*László Kiss declared that he served his sentence and suffered enough* (reversed); *László Kiss declared that the rape never happened* (reversed); *László Kiss resigned;* *α* = 0.64]. We originally included two other items in the measure of moral judgment which were indirectly related to the moral responses to the scale [*The Hungarian Swimming Association stood up for László Kiss, The documents of the László Kiss case was closed* (reversed) *α* = 0.72], however, we omitted them, as they were not the perpetrator’s response to the events. However, it should be noted that these items fit into the scale based on reliability analysis, and the overall patterns are unaffected by its inclusion or removal. Although by the removal the reliability of the scale was lower than conventional standards, this is not necessarily a problem, as an analytical approach suggests “when a measure has other desirable properties, such as meaningful content coverage of some domain…low reliability may not be a major impediment to its use” (Schmitt, 1996, pp. 351–352). We used reversed scoring on all items but one so that a higher mean indicated the more negative moral judgment, using a 7-point scale from 1 = It was completely wrong to 7 = it was completely right.

Participants indicated whether they labeled the case as rape or not using one item and a 7-point scale from 1 = it was certainly not rape to 7 = it was certainly rape.

Participants completed the Hungarian version of the Updated Illinois Rape Myth Acceptance Scale (19 items, α = 0.93; McMahon and Farmer, 2011). The scale was translated into Hungarian with two independent translators, then backtranslated into English, and approved by the original author of the scale, McMahon. The adapted scale had acceptable fit indices on the Hungarian sample (*RMSEA* = 0.05, *CFI* = 0.95, *SRMR* = 0.05; Nyúl et al., 2018).

#### Results

##### Descriptive statistics

Because of the different sampling methods, the two subsamples differed in their perception of the rape case (see Table 1). As expected, the self-selected community sample accepted rape myths less [Levene’s test indicated unequal variances, *F* = 61.09, *p* < 0.001, so degrees of freedom were adjusted from 868 to 639.53, *t*(639.53) = 17.83, *p* < 0.001], cared less about the perpetrator’s success as a swimmer and swimming coach [*t*(868) = 14.25; *p* < 0.001], they evaluated the reaction of the perpetrator as morally less acceptable [Levene’s test indicated unequal variances, *F* = 32.92, *p* < 0.001, degrees of freedom were adjusted from 868 to 708.5, *t*(713.76) = -12.74, *p* < 0.001], labeled the case more clearly as a rape [Levene’s test indicated unequal variances, *F* = 24.13, *p* < 0.001, degrees of freedom were adjusted from 868 to 731.55, *t*(731.55) = -8.75 *p* < 0.001]. Although there were differences between the two samples, we ran all analyses on the combined sample for two reasons. Firstly, we did not have different hypotheses for the two subsamples, and expected the same psychological processes in the biased perception of the rape case. Secondly, combining these samples provided estimates based on more diverse attitudes toward the issue, and thus arguably more representative of the population as a whole than each subsample individually. In order to check that indeed the same psychological mechanisms were present in both subsamples, we ran the mediation analyses an on both samples, and found the same results (see Supplementary Material). Correlations (shown on Table 2) suggested that RMA was negatively associated with moral judgment and rape labeling, and positively associated with perpetrator’s success. Also as expected, rape labeling and moral judgment were positively associated. Perpetrator’s success was negatively associated with moral judgment and rape labeling. These zero-order correlations were in line with our hypotheses and confirmed that it was meaningful to test the mediating role of perpetrator’s success in the connection between RMA and the evaluation of the rape case.

**Table 1:** Descriptive statistics of Study 1.

	Study 1		Self-selected community sample		Student sample	
			
	Mean	*SD*	Mean	*SD*	Mean	SD
Perpetrator’s success	3.34	2.07	2.57	0.08	4.39	0.09
Moral judgment	5.41	1.20	6.25	1.14	5.41	1.20
Rape labeling	5.89	1.51	6.27	1.38	5.38	1.54
RMA	2.05	1.04	1.59	0.76	2.68	1.03


**Table 2:** Correlations between the variables in Study 1.

	1	2	3
(1) Perpetrator’s success			
(2) Moral judgment	–0.36^∗∗∗^		
(3) Rape labeling	–0.23^∗∗∗^	0.46^∗∗∗^	
(4) RMA	0.30^∗∗∗^	–0.27^∗∗∗^	–0.19^∗∗∗^


*^∗∗∗^*p* < 0.001.*

##### Hypothesis testing

We tested whether the connection between RMA and labeling the case as rape as well as moral judgments related to the rape was mediated by the perpetrator’s success using path analyses with the bootstrapping technique, controlling for the effect of gender. As we tested the predictions regarding two outcome variables that were also correlated, we used Structural Equation Modeling in which we relied on maximum likelihood procedure (ML) with 1000 bootstrap samples. We ran the analysis using MPlus Version 7.2 (Muthén and Muthén, 2015). Based on previous theorizing, all variables were allowed to predict the two outcome variables, therefore, the model was fully saturated, allowing us to estimate all path coefficients (for details see Bollen, 1989). Because the model was just identified, the fit indices of the model are not informative (df = 0).

**FIGURE 1:**
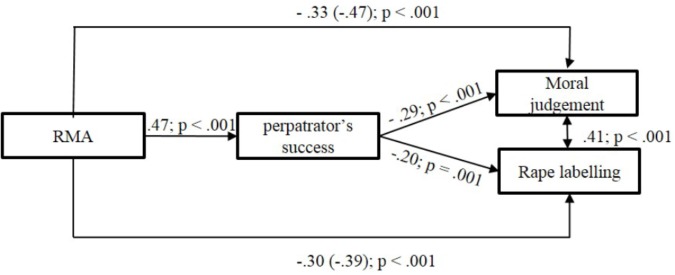
Standardized path model of the direct and indirect effects on Moral judgment and Rape labeling in Study 1.

The result of the path analysis revealed that higher RMA predicted higher importance of perpetrator’s success, lower moral judgment and rape labeling directly, and higher perpetrator’s success predicted lower moral judgment and rape labeling. As expected, RMA did not only directly predict rape labeling and moral judgment, but this effect was mediated by the importance of the perpetrator’s success. As expected, rape labeling and moral judgment were positively associated (see Figure 1 for a visual presentation of the significant paths and see Table 3 for the direct effects – variance is explained by the RMA via the mediator – and indirect effects – variance is explained only by RMA).

**Table 3:** Standardized estimates of direct and indirect effects on moral judgment and rape labeling in Study 1.

	Standardized β	95% CI	*SE*	*p*
RMA → Moral judgment (total effect)	–0.47	[-0.55; -0.39]	0.04	<0.001
RMA → Perpetrator’s success → Moral judgment (indirect effect)	–0.14	[-0.17; -0.10]	0.02	<0.001
RMA → Moral judgment (direct effect)	–0.33	[-0.42; -0.24]	0.04	<0.001
RMA → Rape labeling (total effect)	–0.39	[-0.47; -0.31]	0.04	<0.001
RMA → Perpetrator’s success → Rape labeling (indirect effect)	–0.09	[-0.13; -0.06]	0.02	<0.001
RMA → Rape labeling (direct effect)	–0.30	[-0.39; -0.21]	0.05	<0.001


*95% Confidence intervals were calculated with 1000 bootstrap samples.*

#### Discussion of Study 1

We hypothesized that higher RMA would predict less severe moral judgment of the case and lower likelihood of labeling the case as rape, and this connection would be mediated by giving importance to the positive perception of the perpetrator as a swimmer and swimming coach. Our results supported this hypothesis. We also hypothesized that consideration for the perpetrator’s success would be predicted by RMA which was also supported by ours. These results are in line with Eyssel and Bohner (2011) findings that participants with higher RMA would use any information to judge the actual rape scenario. Our results are also in line with Knight et al.’s (2001) findings that a perpetrator’s success can function as an excuse in the evaluation of a rape case. We also found that participants with higher RMA judged the perpetrator’s response morally less harshly and labeled the case less as a rape, both of which are in line with previous findings (see Burt and Albin, 1981; Norris and Cubbins, 1992; Cowan and Curtis, 1994). In sum, in the uncertain context of this specific rape scandal, RMA and the biased perception of the perpetrator predicted whether participants labeled the incident – that happened 55 years earlier and even before the perpetrator was a well-known and celebrated person – as rape, and how they judged consequent reactions morally. Participants used perception of the perpetrator to justify their views in the moral evaluation of the consequences. This finding suggests that as long as there is room for relativizing a rape case, excusing the perpetrator, and blaming the victim, RMA plays an important role in predicting the cognitive bias in the perception of the perpetrator that in turn can predict different evaluations of the case.

### Study 2

After data was collected for Study 1, there was an unexpected turn of events, as the victim turned out to be alive, and came forward with the story of her rape. Following her appearance, the swimming coach publicly admitted the crime. The fact of rape became undisputable. We could therefore compare the effect of the biased perception of the perpetrator of the same rape case when it was uncertain and when it became undisputable. Building on the findings in Study 1 about the moral judgment of the perpetrator’s reactions and labeling the case as rape, we expected that the biased perception would no longer have an effect on labeling the case as rape, while we hypothesized that RMA would continue to predict the moral judgment of the case, and this connection would be mediated by the perpetrator’s perception as a successful swimmer/swimming coach (Burt and Albin, 1981; Norris and Cubbins, 1992; Peterson and Muehlenhard, 2004).

#### Participants

Participants were university students who completed the questionnaire for course credit (*N* = 105; 29.5% male, *M*_age_ = 21.78; *SD*_age_ = 3.98).

#### Procedure and Measures

We relied on the same procedure and the same measures as in the Study 1 with minor adjustments to the new context. We extended the summary with the information that the victim was alive and László Kiss admitted his crime and apologized to her. Respondents rated how important it was that the perpetrator was a successful swimmer/swimming coach by two items (*r* = 0.75, *p* < 0.001), and labeled the case whether they considered it a rape or not, and completed the RMA Scale (19 items, α = 0.92; McMahon and Farmer, 2011). We added one item to the *moral judgment* *of the perpetrator’s response* scale (*László Kiss apologized to Zsuzsanna Takáts*) so it became a 4-item scale (α = 0.47). Again we used 7-point scales for all items.

#### Results

##### Descriptive statistics

In contrast to the results of Study 1 (see Table 4), most participants agreed that it was rape what happened [*M*_Study1_ = 5.89, *SD*_Study1_ = 1.51, *M*_Study2_ = 6.17, *SD*_Study2_ = 1.46 Levene’s test indicated unequal variances, *F* = 5.62, *p* = 0.018, degrees of freedom were adjusted from 973 to 132.51, *t*(132.51) = 1.84 *p* = 0.068] but people did not judged the moral response more negatively [*M*_Study1_ = 5.89, *SD*_Study1_ = 1.19, *M*_Study2_ = 5.89, *SD*_Study2_ = 1.19, *F* = 2.41, *p* = 0.121, *t*(973) = -8.19, *p* = 0.029]. Zero-order correlations were highly similar to Study 1, variables were associated in the expected direction, higher rape myths acceptance was associated with higher perceived success of the perpetrator, labeling the case as rape and moral judgment, and these variables were also positively associated (see Table 5).

**Table 4:** Descriptive statistics of Study 2.

	Mean	*SD*
Perpetrator’s success	3.59	1.98
Moral judgment	5.91	1.03
Rape labeling	6.17	1.46
RMA	2.67	1.16


**Table 5:** Correlations between the variables in Study 2.

	1	2	3
(1) Perpetrator’s success			
(2) Moral judgment	–0.10		
(3) Rape labeling	–0.03	0.49^∗∗∗^	
(4) RMA	0.36^∗∗∗^	–0.44^∗∗∗^	–0.08


*^∗∗∗^*p* < 0.001.*

##### Hypothesis testing

Similarly to Study 1 we used path analysis with the bootstrapping technique controlling the effect of gender in the analysis to test our hypothesis about the mediating role of the perpetrator’s success in labeling the case as rape and judging the reactions morally. Again, we used Structural Equation Modeling with the maximum likelihood procedure (ML) with 1000 bootstrap samples. In contrast to the model of Study 1, but in line with our hypothesis, this model was not fully saturated, but offered good fit to the data (*χ^2^* = 61.99 (*df* = 9), *Comparative Fit Index (CFI)* = 1.00, *Tucker-Lewis index (TLI)* = 1.00, *Root Mean Square Error of Approximation* (*RMSEA)* = 0.000, *90% confidence interval* [0.00; 0.08]).

**FIGURE 2:**
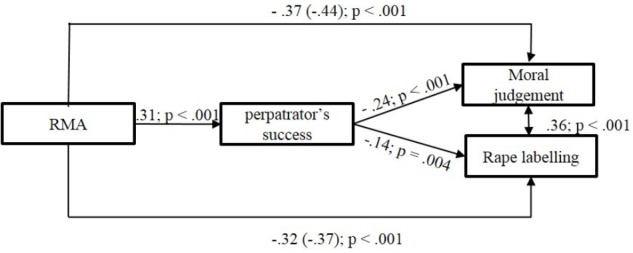
Standardized path model of the direct and indirect effects on Moral judgment and Rape labeling in Study 2.

We found that neither RMA, nor the perpetrator’s perceived success predicted labeling the case as rape. However, RMA was a significant predictor of moral judgment, but the perpetrator’s perceived success did not mediate this connection, so only the direct path was significant. (For a visual presentation of the path model see Figure 2).

#### Discussion of Study 2

We hypothesized that the perception of the perpetrator’s success would no longer have an effect on labeling the case as rape as the label became undisputable after it was admitted by the perpetrator. However, we expected that RMA would continue to predict the moral judgment of the case and this would continue to be mediated by perceiving the perpetrator as a successful swimmer/swimming coach. The hypothesis was only partly supported. In line with the hypothesis, we found that indeed RMA and the perceived success of the perpetrator became irrelevant in labeling the case as rape. This finding is in line with Bongiorno et al.’s (2016) study that found that circumstances, such as the group membership of the perpetrator do not matter if the rape is perceived as stereotypical, that is, when people tend to accept it as real. However, the moral judgment of the responses to the scandal were still affected by RMA, that is, higher acceptance of rape myths predicted less harsh moral judgments. This finding suggests that participants with higher RMA could not or did not deny the case more than those with lower RMA, but continued to judge the situation less severely. This finding can be interpreted in a way that people with higher acceptance of rape myth judge rape as a less severe criminal act in general (see Norris and Cubbins, 1992; Frese et al., 2004).

## General Discussion

The aim of our study was to examine whether people with higher RMA are more likely to use the positive public perception of a famous person as an excuse for committing rape or even for labeling the case as rape regardless of the fact that the rape happened even before the person became famous. We also wanted to show whether these psychological processes can be identified when it comes to an actual case that people learned about in the media as opposed to the evaluation of cases presented in lab studies using vignettes. Furthermore, this real-life story with high public awareness allowed us to compare judgments when the case was somewhat more uncertain and when the same case became indisputable.

Our findings about the role of RMA in believing the perpetrator’s denial and taking into account that he was a successful person in the uncertain case, supplemented previous research suggesting that irrelevant factors can affect the evaluation of a rape case (e.g., George and Martínez, 2002; Harrison et al., 2008; Bal and van den Bos, 2010; Bongiorno et al., 2016). Our results therefore also confirm that RMA functions as biased information processing, as people seek consistent information to confirm their preexisting beliefs (Eyssel and Bohner, 2011; Süssenbach et al., 2012). Indeed, we found that RMA affected the evaluation of a real-life rape case, and the current perception of the perpetrator as a successful person functioned as an excuse, especially when RMA was high.

On top of identifying the biased perception of the perpetrator and the biased evaluation of the rape case connected to RMA, the turn of events provided a unique opportunity to also examine whether these connections changed when the fact of rape became indisputable. We found that RMA still had a direct effect on moral judgments, but it no longer predicted rape labeling. Furthermore, the perpetrator’s success did not affect moral judgment or rape labeling anymore. Although these results suggest that biased information processing had a more powerful effect on the evaluation of the case when the rape was uncertain, previous attitudes about rape continued to affect moral judgments even when the case was indisputable.

These findings support Eyssel and Bohner (2011) theory that rape myths function as cognitive schemas and therefore predispose the perception of a rape case. In line with previous findings, RMA directly predicted rape labeling and moral judgments, but more importantly for people with high RMA the celebrity status of the perpetrator was also important in judging the case (Eyssel and Bohner, 2011; Süssenbach et al., 2012). This finding fits with previous research about the connection between the perpetrator’s celebrity status and the evaluation of the rape case (Knight et al., 2001; Loeffler and Lawson, 2002; Devine and Caughlin, 2014). Similarly to Bongiorno et al. (2016) study, in which they found that group membership of the perpetrator was taken into account only in non-stereotypical cases of rape, we found that when rape was uncertain, people relied on irrelevant information more to justify their evaluations, especially when it was in line with their previous attitudes about rape.

## Limitations and Future Directions

Although our research about an actual rape case provided a unique opportunity to test the effect of RMA, our data collection had some limitations. For example, we used a cross-sectional research design, and therefore, we could not identify how individual respondents’ beliefs changed between the two times of the data collection.

Furthermore, our results supported the association between the constructs that we included in our studies, but could not identify their causal connections, and therefore offer only limited information about potential areas of interventions. Previous research mostly treated RMA as a stable construct, nevertheless, there is some evidence that RMA can be influenced by situational variables such as perceived social norms (Bohner et al., 2006). Despite this general treatment of RMA as a stable attitudinal dimension, we cannot rule out the possibility that perceived success of the perpetrator could potentially increase RMA. Such a connection would imply that the causal connection between the variables would be the opposite of what was tested in the current research. Therefore, future research should test the effect of celebrity status using an experimental design for example by its direct manipulation (see, Knight et al., 2001) to establish the causal connection between these two variables. Future studies could also test the moderating effect of RMA to find out whether celebrity status of the perpetrator affects people with high and low RMA differently.

Before the items of our study variables, we presented some facts related to the scandal to the participants. Although the case was widely known after the scandal broke out, we did not examine participants’ prior knowledge of the case. The different or the lack of knowledge could have affected the way the participants thought about the victim and the perpetrator, and through this the evaluation of the case.

The strength of our research is its the ecological validity and the fact that we collected data at the time the scandal broke out, therefore, we did not have to rely on people’s memory of the event, but measure their immedate responses. However, this created a caveat for the study. The central variable of our research was the perception of the perpetrator’s success in the evaluation of the rape case. As this has not been measured in previous studies, we could not rely on a validated or previously used measure for this construct. Therefore, we chose the most straightforward option of directly asking respondents about how important it was for them in the evaluation of the rape case that the perpetrator was a successful athlete and coach. However, we acknowledge that this response may have been affected by respondents’ perception of his success. As we did not collect data about the evaluation of his success in general, we have no way to know whether individual differences in the acknowledgment of his personal success may have affected how important they considered this information in evaluating the rape case.

Although in this research we examined the impact of the perpetrator’s success on the evaluation of a rape case, other factors could have been included in our research contributing to the overall evaluation of the case, such as the historical context of the crime, the different social norms regarding rape at the time and the time which has passed since it occurred. Future research should focus on extending the study to the effects of other factors.

In the Study 1. we used two different convenience samples: a self-selected community sample and a university student sample to test our hypotheses. We used the community sample that we recruited in social media groups relevant to the topic of rape to understand the decision-making processes of people who have a stronger motivation to express their opinion and consequently to influence public opinion on these issues. We supplemented this self-selected community sample by students to also see how more naïve participants formulate their opinion on the case. Although collecting two subsamples we had a more diverse sample, the use of these samples have limitations in terms generalizability of our findings to the broader population.

For the purpose of this research, we used the example of a swimming coach because this was the example presented to us by real-life events. However, based on this case, we cannot be sure that patterns would be exactly the same if the perpetrator was a different kind of celebrity. Future studies should consider testing the effect of different types of celebrities, especially taking into account that celebrities represent different types of role models. Although actors and musicians may be more popular and admired than successful sport persons, however, from a moral perspective their lifestyle and acts may be more harshly evaluated and considered less normative than that of a sportsperson (Bricheno and Thornton, 2007). Furthermore, we can distinguish between different kinds of success, such as success based on high social status and success based on competence or hard work. Future studies could focus on whether these two sources success have different effects on the evaluation of the perpetrator and the rape case.

An additional asset of this study is that we conducted our research in an underrepresented region of social psychological research, and especially of research on rape and rape myths. This region is not simply underrepresented in these research areas, but also the level of sexism is higher and gender equality is lower in Hungary than in the United States or in Western Europe (World Economic Forum, 2016). Therefore, our findings could show that the connection between RMA, the biased perception of the perpetrator and the evaluation of a rape case is also pronounced in a cultural context in which sexism appears in more overt, more hostile and more explicit ways than in the most commonly studied countries, such as the United States.

Our research has shown that situational factors are highly relevant in the evaluation of an uncertain rape. This finding is important because most rape cases are uncertain and counter-stereotypic in reality and do not fit the assumptions that rape is committed by strangers using physical threat to rape. Furthermore, it is also common that victims do not report the case. This helps us understand why people (a) do not necessarily label every rape case as rape, (b) do not consider every rapist a criminal, (c) seek belief consistent information that help them excuse the perpetrator, (d) perceive the act as a milder form of misbehavior, (e) blame the victim, and (f) minimize the impact of the rape on the victim. All of this can be done without changing their overall opinion about rape as a reprehensible crime. Therefore, in counter-stereotypical and uncertain contexts, irrelevant information, such as the perpetrator’s success can seriously affect the evaluation of the event as rape or the evaluation of its severity especially for people who tend to accept rape myths. In line with the findings of Eyssel and Bohner (2011), people view rape cases according to their previous attitudes, and tend to stick to these attitudes especially if they believe that they have more information about the case, such as knowledge about the perpetrator. Our findings indicate that RMA can function as such pre-existing attitude toward rape and motivate people to seek consistent information and give more weight to them in evaluating the case.

In conclusion, we found that people used positive information about the perpetrator in evaluating an uncertain rape case. This understanding highlights the responsibility of rape case reporting, clearly indicating that offering additional, irrelevant, but positive information about the perpetrator can increase victim blaming and excusing the victim especially if the information meets people’s preexisting beliefs about rape. This is not only relevant for the evaluation of individual cases, but also because media reports of rape affect public opinion and the normative context in which all rape cases are evaluated.

## Data Availability Statement

The datasets for this study can be found in the Open Science Foundation https://osf.io/7ayg9/?view_only=3edf904804d046158 53b04f4f9a451f2.

## Ethics Statement

This study was carried out in accordance with the recommendations of Ethical Guidelines and Ethical Committee of Eötvös Loráns University with written informed consent from all subjects. All subjects gave written informed consent in accordance with the Declaration of Helsinki. The protocol was approved by the Ethical Committee of Eötvös Loráns University.

## Author Contributions

BN contributed the research idea and the general design of the studies. She was responsible for data analysis and the preparation of the manuscript. AK helped with the design of the studies, data analysis, and the preparation of figures and tables. She also contributed to the preparation of the manuscript. ME helped with the pathway analysis and with the results section. MS helped with the general design of the studies.

## Conflict of Interest Statement

The authors declare that the research was conducted in the absence of any commercial or financial relationships that could be construed as a potential conflict of interest.
