# Supplementary material for: Perception of a Perpetrator as a Successful Person Predicts Decreased Moral Judgment of a Rape Case and Labeling it as Rape
Source: Front Psychol. 2018 Dec 11;9:2555. doi: 10.3389/fpsyg.2018.02555 (PMC6297382; doi:10.3389/fpsyg.2018.02555)
Supplement: Supplementary file 1 [file Table_1.pdf]

Table S1.

Standardized estimates of direct and indirect effects on moral judgement and rape labelling in Study 1 Self-selected community sample

	Standardized β	95% CI	SE	p
RMA → Moral judgement (total effect)	-.44	[-.58 ; -.30]	.08	< .001
RMA → Perpetrator's success → Moral judgement (indirect effect)	-.07	[-.11 ; -.03]	.02	< .001
RMA → Moral judgement (direct effect)	-.37	[-.51 ; -.23]	.07	< .001
RMA → Rape labelling (total effect)	-.37	[-.51 ; -.23]	.08	< .001
RMA → Perpetrator's success → Rape labelling (indirect effect)	-.04	[-.08 ; -.01]	.02	.008
RMA → Rape labelling (direct effect)	-.32	[-.46 ; -.19]	.07	< .001

Note: 95% Confidence intervals were calculated with 1000 bootstrap samples.

Table S2.

Standardized estimates of direct and indirect effects on Moral judgement and Rape labelling in Study 1 Student sample

	Standardized β	95% CI	SE	p
RMA → Moral judgement (total effect)	-.30	[-.41; -.19]	.06	< .001
RMA → Perpetrator's success → Moral judgement (indirect effect)	-.10	[-.14; -.05]	.02	< .001
RMA → Moral judgement (direct effect)	-.20	[-.32 ; -.08]	.06	.001
RMA → Rape labelling (total effect)	-.22	[-.33 ; -.10]	.06	< .001
RMA → Perpetrator's success → Rape labelling (indirect effect)	-.06	[-.10 ; -.02]	.02	.003
RMA → Rape labelling (direct effect)	-.16	[-.28 ; -.04]	.06	<.010

Note: 95% Confidence intervals were calculated with 1000 bootstrap samples.
